# Altered gut microbiota and microbial biomarkers associated with chronic kidney disease

**DOI:** 10.1002/mbo3.678

**Published:** 2018-08-07

**Authors:** Hengzhong Lun, Weihua Yang, Shuping Zhao, Meijie Jiang, Mingjie Xu, Fenfen Liu, Yunshan Wang

**Affiliations:** ^1^ Medical Research & Laboratory Diagnostic Center Jinan Central Hospital Affiliated to Shandong University Jinan Shandong China; ^2^ The Department of Clinical Laboratory Taian City Central Hospital Taian Shandong China; ^3^ The Department of Nephrology Taian City Central Hospital Taian Shandong China

**Keywords:** chronic kidney disease, gut microbiota, hemodialysis, intestinal dysbiosis, microbial biomarker

## Abstract

The present study aimed to determine the differences in gut microbiota between patients with chronic kidney disease (CKD) and healthy controls (HC) and search for better microbial biomarkers associated with CKD. The 16S rRNA gene sequencing approach was used to investigate the differences in gut microbiota between the CKD and HC groups. The study found that 12 phylotypes were overrepresented in the CKD group and 19 in the HC group at the genus level. Furthermore, genera *Lachnospira* and *Ruminococcus_gnavus* performed the best in differentiating between HC and CKD populations. In addition, this novel study found that the genera *Holdemanella*,* Megamonas*,* Prevotella* 2*, Dielma*, and *Scardovia* were associated with the progression of CKD and hemodialysis. In conclusion, the composition of gut microbiota was different in CKD populations compared with healthy populations, and *Lachnospira* and *R._gnavus* were better microbial biomarkers. In addition, five phylotypes, including *Holdemanella*,* Megamonas*,* Prevotella*2, *Dielma*, and *Scardovia*, served as an indicator of the progression of CKD and hemodialysis. However, large‐scale prospective studies should be performed to identify the reliability of the set of these phylotypes as biomarkers.

## 1 INTRODUCTION

Chronic kidney disease (CKD) is characterized by a reduced glomerular filtration rate and progressive impaired renal function. It has become a global public health issue with an estimated prevalence of 8%–16% (Jha et al., 2013). Worldwide, diabetes mellitus, hypertension, and glomerulonephritis are the common causes of CKD. Patients with CKD experience several complications including increased blood pressure, anemia, acute kidney injury, cardiovascular mortality, and accumulation of high levels of uremic toxins.

More than 2,000 species of commensal bacteria live in the intestinal tract in a natural balance. They constitute a dynamic and symbiotic ecosystem that interacts with the host metabolism (Bourlioux, Koletzko, Guarner, & Braesco, 2003; Dunne, 2001; Hooper & Gordon, 2001; Iannitti & Palmieri, 2010). A large number of recent studies demonstrated the association of gut microbiota with many diseases including obesity, type 2 diabetes, and nonalcoholic fatty liver disease (Abu‐Shanab & Quigley, 2010; Qin et al., 2012; Ridaura et al., 2013). Recent studies have suggested that the gut microbiota is one of the pathogenic factors in kidney disease (Anders, Andersen, & Stecher, 2013). Vaziri and his colleagues used the 16S rRNA gene PhyloChip technique to demonstrate that uremia profoundly altered intestinal microbial flora (Vaziri, Wong, et al., 2013). Furthermore, some studies investigated the potential of intestinal microbial flora as microbial biomarkers for the noninvasive diagnosis and selection of an appropriate personalized treatment (Pascal et al., 2017; Sommer et al., 2017). However, it remains unclear which microbial biomarkers are more appropriate for CKD.

Therefore, this study was performed to explore the differences in gut microbiota between patients with CKD and healthy controls (HC) and further search more appropriate microbial biomarkers associated with CKD.

## 2 MATERIAL AND METHODS

### 2.1 Study population

This study included 49 patients with CKD (37 males and 12 females aged 54 ± 14 years) and 24 HC (16 males and eight females aged 56 ± 9 years) (age, Student *t* test, *p *=* *0.303). All enrolled participants signed the informed consent. The study was reviewed and approved by the Medical Ethics Committee of Taian City Central Hospital, Taian, China. Of the 49 patients with CKD, 13 underwent hemodialysis and the remaining were never treated with dialysis. Patients with malignancy, pregnancy, acute or chronic infections, chronic inflammatory bowel disease, and celiac disease were excluded. All participants in this study did not take any antibiotics, probiotics, prebiotics, or symbiotics during the 3 months before fecal samples were collected. Stool samples from enrolled participants were collected in a sterile plastic cup and stored at −80°C for further microbiome analysis.

### 2.2 Microbiota analysis

The V3–V4 region of the 16S rRNA gene was amplified and sequenced on the Illumina Hiseq System (PE250) to profile the microbial composition of fecal samples. The forward primer was 341F: CCTAYGGGRBGCASCAG, and the reverse primer was 806R: GGACTACHVGGGTWTCTAAT. All polymerase chain reactions were performed in 20‐μl reaction mixtures with 4 μl of 5 ×  FastPfu Buffer, 2 μl of 2.5 mM dNTPs, 0.5 μM forward and reverse primers, 0.4 μl of FastPfu Polymerase, and 10 ng template DNA. Thermal cycling consisted of initial denaturation at 95°C for 5 min, 27 cycles of denaturation at 99°C for 30 s, annealing at 55°C for 30 s, elongation at 72°C for 45 s, and maintenance at 72°C for 10 min. The library was constructed and sequenced on an Illumina HiSeq platform. Sequences were grouped into operational taxonomic units (OTUs) using the Usearch (version 7.1 http://drive5.com/uparse/) algorithm and assigned to the same OTU with a distance‐based similarity of ≥97% (Edgar, 2010). Assigned sequence reads were used to assess the differences in taxonomic abundances between CKD and HC at the phylum level. The measurements of beta diversity were performed with the principal component analysis (PCA) based on the Euclidean distance and nonmetric multidimensional scaling (NMDS) based on the UniFrac distance. PCA and NMDS plots were generated using the R package vegan. The significantly differential taxa between groups were determined using LEfSe (http://huttenhower.sph.harvard.edu/galaxy/root?tool_id=lefse_upload), which performs a nonparametric factorial Kruskal–Wallis rank‐sum test followed by the linear discriminant analysis (LDA) coupled with measurements to assess the effect size of each differentially abundant taxon, and the threshold of the LDA was set to 2 (Segata et al., 2011).

The ability of the microbial markers to differentiate between HC and CKD was evaluated using the area under the receiver operating characteristic (ROC) curve. The phylotypes detected in hemodialysis samples and HC group were discovered by evaluating the data from high‐throughput sequencing.

### 2.3 Statistical analysis

The statistical analysis of quantitative data and ROC was conducted with the Student *t* test using SPSS 16.0. A *p* value less than 0.05 was considered statistically significant.

## 3 RESULTS

The gut microbiota of patients with CKD and HC were investigated using the high‐throughput sequencing of the V3–V4 region of the 16S rDNA gene, and a total of 2,754,607 reads were sequenced.

### 3.1 Bacterial microbiota composition

**Figure 1:**
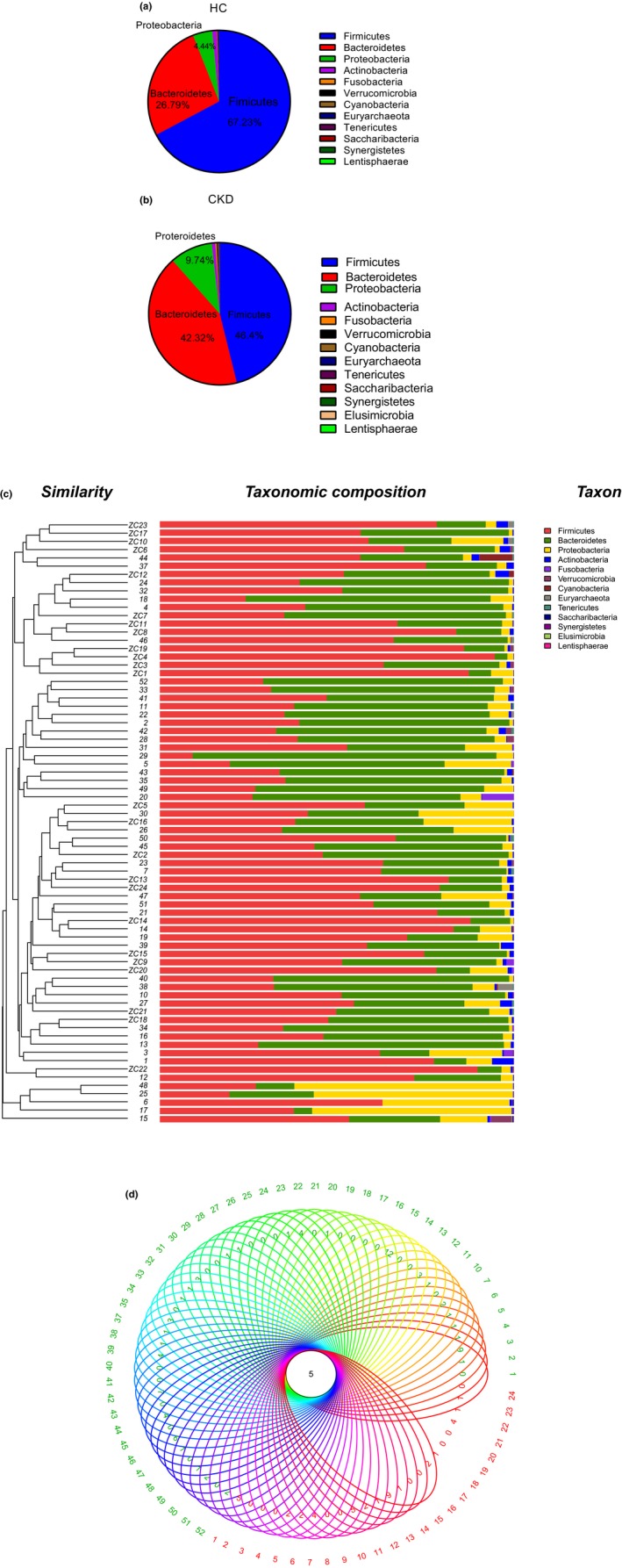
Taxonomic analysis of gut microbiota from 16S rRNA sequencing. (a) Composition of gut microbiota (phylum) of HC. (b) Composition of gut microbiota (phylum) of CKD. (c) Relative abundance of microbial community at the phylum level in fecal samples collected from both the CKD and HC groups. (d) Venn diagram of fecal microbiota at the OTU level. Each ellipse represents one sample. Red represents HC, whereas green represents CKD

In this study, most of the bacteria of fecal samples collected from both CKD and HC groups belonged to the phyla *Firmicutes*,* Bacteroidetes*,* Proteobacteria*, and *Actinobacteria* (98.2% and 98.46%, respectively) (Figure 1a,b). The relative abundance of the dominant main phyla *Firmicutes*,* Bacteroidetes*, and *Proteobacteria* between CKD and HC groups significantly changed. The CKD group had a significantly higher abundance of *Bacteroidetes* and *Proteobacteria* and lower abundance of *Firmicutes* compared with the HC group (Student *t* test, *p *=* *0.002, 0.03, and 0.000, respectively). The clustering analysis also showed that the CKD group differed from the HC group at the phyla level (Figure 1c). The Venn diagram represented shared/unique OTUs in the gut microbiota of the CKD and HC groups (Figure 1d).

### 3.2 Bacterial microbiota diversity analysis

**Figure 2:**
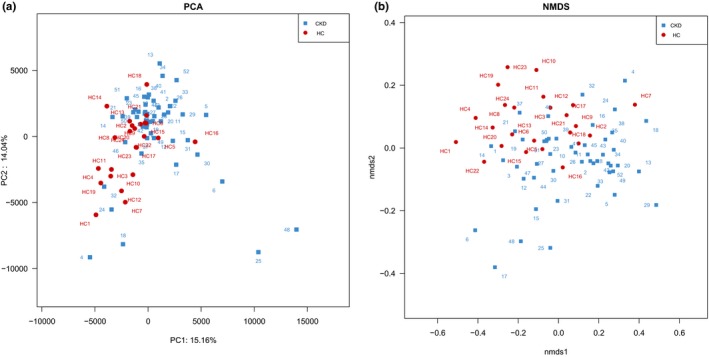
(a) Visualization of the PCA analysis based on the Euclidean distance. (b) Nonmetric multidimensional scaling (NMDS) plot of microbial communities, based on the OUT level, derived from fecal samples of HC (red) and patients with CKD (blue)

The beta diversity of the samples was evaluated using PCA based on the Euclidean distance to investigate the differences in the structure of gut microbiota (Figure 2a). The result revealed that the gut microbiota of patients with CKD were distinct from those of the HC with the first two principal component scores 15.16% and 12.24%, respectively. NMDS based on UniFrac distance also showed a separation trend of the HC and CKD groups at the OTU level (Figure 2b).

### 3.3 Differences in gut microbiota between the CKD and HC groups

**Figure 3:**
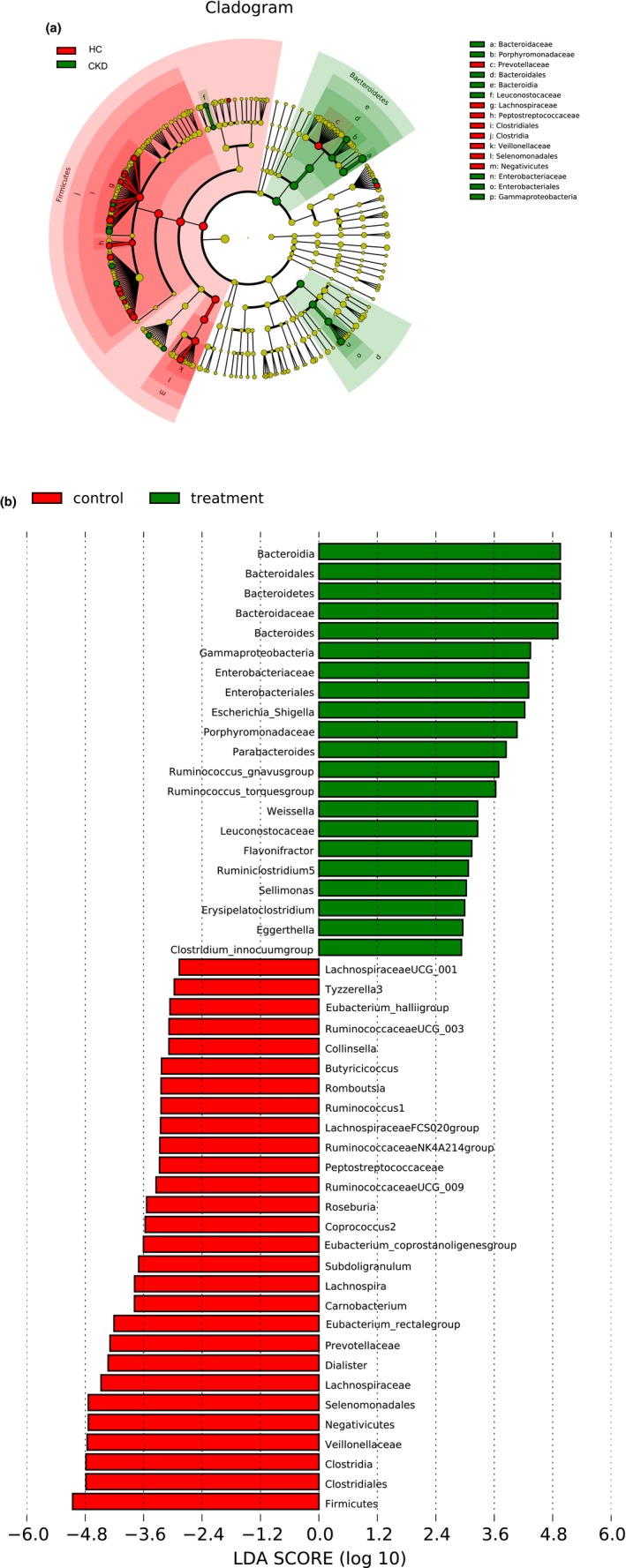
(a) Cladogram showing the most differentially abundant taxa identified by LEfSe. Red indicates clades enriched in the HC group, whereas green indicate clades enriched in the CKD group. (b) Comparisons of gut bacteria between the HC and CKD groups. The histogram shows the LDA score computed for genera differentially abundant between groups and identified using LEfSe

The differences in gut microbiota between the CKD and HC groups were determined using the Linear discriminant analysis Effect Size (LEfSe). The cladogram generated from the LEfSe analysis showed the most differentially abundant taxa enriched in microbiota from the CKD or HC group (Figure 3a). The histogram indicated that a difference of 49 phylotypes was observed between the CKD and HC groups (Figure 3b). A total of 31 phylotypes were different at the genus level, and they might serve as biomarkers to differentiate between CKD and HC populations. Among the 31 phylotypes, 12 were more abundant in the CKD group, including *Bacteroides, Escherichia_Shigella, Parabacteroides, Ruminococcus_ gnavus, Ruminococcus torques, Weissella, Flavonifractor, Ruminiclostridium5, Sellimonas, Erysipelatoclostridium, Eggerthella*, and *Clostridium_innocuum*. Furthermore, 19 phylotypes were more abundant in the HC group, including *Dialister, Eubacterium rectale, Carnobacterium, Lachnospira, Subdoligranulum, Eubacterium_coprostanoligenes, Coprococcus2, Roseburia, RuminococcaceaeUCG_009, RuminococcaceaeNK4A214, LachnospiraceaeFCS020, Ruminococcus1, Romboutsia, Butyricicoccus, Collinsella, RuminococcaceaeUCG_003, Eubacterium_halliigroup, Tyzzerella3*, and *LachnospiraceaeUCG_001*.

### 3.4 Comparison of microbial biomarkers

**Figure 4:**
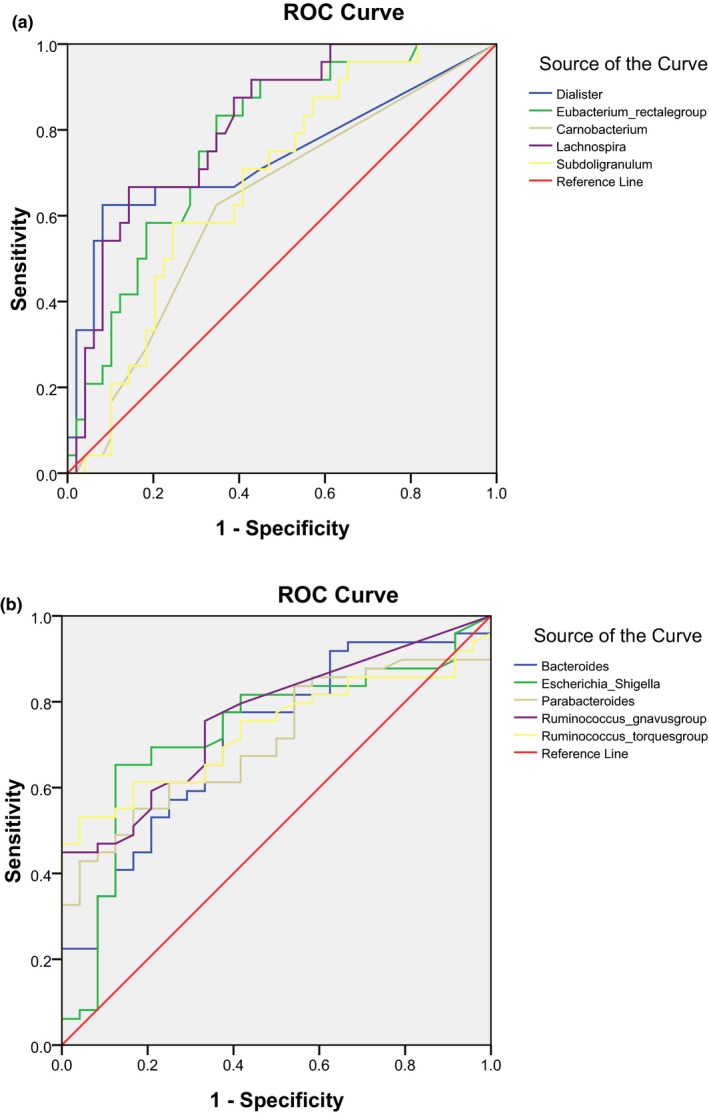
ROC curves for microbial biomarkers. (a) ROC curves of five phylotypes with the highest LDA score at the genus level in the HC group. (b) ROC curves of five phylotypes with the highest LDA score at the genus level in the CKD group. A higher curve generally indicates a better method. AUC statistic summarizes the trade‐offs across the varied sensitivity/specificity range

ROC curves of five phylotypes with the five highest LDA scores at the genus level in the HC and CKD groups and their area under the ROC curve (AUC) values were obtained to explore the ability of the gut microbiome to differentiate between the HC and CKD groups. Out of the five phylotypes tested, *Lachnospira* achieved the best performance in the HC group (AUC = 0.813; 95% CI: 0.713–0.912; *p *=* *0.000) (Figure 4a) and *Ruminococcus_gnavus* was the best in the CKD group (AUC = 0.764; 95% CI: 0.656–0.873; *p *=* *0.000) (Figure 4b).

### 3.5 Microbial markers associated with the progression of CKD and hemodialysis

The data from high‐throughput sequencing were analyzed to explore microbial species associated with the progression of CKD and hemodialysis. At the genus level, the genera *Holdemanella*,* Megamonas*, and *Prevotella2* were not detected in the HD samples, and the rate of their detection decreased in the CKD group compared with the HC group. The genera *Dielma* and *Scardovia* were not detected in all the HC samples (Table**** 1).

**Table 1:** Detection rate of bacteria at the genus level

Bacteria	HD	Non‐HD	HC
*Holdemanella*	0%	11.76%	33.33%
*Megamonas*	0%	14.71%	45.83%
*Prevotella2*	0%	5.88%	29.17%
*Dielma*	31%	29.41%	0.00%
*Scardovia*	23%	2.94%	0.00%

## 4 DISCUSSION

This study used the high‐throughput sequencing of the fecal microbiota of patients with CKD and HC to investigate the alteration of intestinal microbiota in patients with CKD. Significant differences in the composition of gut microbiota were found between the CKD and HC groups. At the phylum level, a significantly increased prevalence of *Bacteroidetes* and *Proteobacteria* and a reduced prevalence of *Firmicutes* were found in patients with CKD. Gut microbiota are vital in the progression of CKD. The fermentation of amino acids tyrosine and tryptophan obtained from food by intestinal microbiota generates *p*‐cresol and indole, respectively, which are further metabolized to generate *p*‐cresyl sulfate and *p*‐indoxyl sulfate in the liver after absorption. These toxins are eliminated mainly by tubular secretion in the kidneys. Their increased concentration is associated with renal impairment and advancing CKD (Poesen, Meijers, & Evenepoel, 2013).

The relationship between the human microbiome and kidney disease is bidirectional. CKD also affects the structure of gut microbiota and contributes to symbiosis. Studies on uremic rats showed marked azotemia, systemic oxidative stress, and marked depletion of the key protein constituents of the epithelial tight junction (claudin‐1, occludin, and ZO1) in the stomach, jejunum, and ileum (Vaziri, Yuan, Nazertehrani, Ni, & Liu, 2013). Many factors are associated with CKD, including frequent use of antibiotics (Jakobsson et al., 2010), decreased consumption of dietary fibers (Kalantar‐Zadeh, Kopple, Deepak, Block, & Block, 2002), metabolic acidosis, slow colonic transit, intestinal wall edema, volume overload with intestinal wall congestion, and oral iron intake (Fouque et al., 2014; Nakao, 2012; 2005). Many of these factors lead to the translocation of bacteria and microbial dysbiosis. Elevated levels of gut fluid urea and uric acid and reduced levels of fiber‐derived short‐chain fatty acids, which contribute to uremia‐induced microbial dysbiosis (Wong et al., 2014), may be the causal factors for microbial dysbiosis in patients with CKD. When the kidney function is insufficient, urea is secreted into the gastrointestinal tract, hydrolyzed by the expansion of urease‐possessing bacteria, and results in the production of large quantities of ammonia, which contributes to systemic inflammation by disrupting the gut epithelial tight junction (Kang, 1993; Vaziri, Duresmith, Miller, & Mirahmadi, 1985; Vaziri, Yuan, & Norris, 2013; Wong et al., 2014). In addition, the intestinal dysbiosis of microbiota may be due to iatrogenic causes. Hemodialysis can contribute to recurrent regional ischemia and systemic circulatory stress, which may also damage the mechanical barrier of the gut (Ding & Li, 2003).

At the genus level, 12 phylotypes were overrepresented in the CKD group and 19 in the HC group. These 31 phylotypes might serve as biomarkers to differentiate between CKD and HC populations. Furthermore, the ability of the gut microbiome with a higher LDA score to differentiate between the HC and CKD groups was assessed. It was found that *Lachnospira* and *Ruminococcus_gnavus* achieved the best performance, respectively, in the two groups. Previous studies showed that the decrease in the abundance of genus *Lachnospira* in the gut microbiome was associated with many diseases. Stiemsma et al. (2016) reported that the abundance of genus *Lachnospira* (L) decreased in children with asthma, and opposing shifts in the relative abundance of *Lachnospira* and *Clostridium neonatale* (C) in the first 3 months of life were associated with preschool‐age asthma, and the L/C ratio might serve as a potential early‐life biomarker to predict asthma development. Wang et al. (2017) found a significant loss of *Lachnospira* in pediatric patients with Crohn's disease prior to infliximab treatment. In the present study, the relative abundance of *Ruminococcus_gnavus* was higher in the CKD group. A study reported that the high consumption of red meat, which increased the risk of colorectal cancer, lowered the abundance of *Ruminococcus_gnavus* (Le Leu et al., 2015).

In addition, this novel study found that *Holdemanella*,* Megamonas*,* Prevotella2, Dielma*, and *Scardovia*, which had some association between the detection rate and advancing CKD, had the potential to serve as indicators of the progression of CKD and HD. Moreover, the five phylotypes were used to study the mechanism of interaction between intestinal flora and CKD. Stein (2015) reported that the prevalence of *Prevotella* significantly reduced in mice with renal failure, which was in line with the findings of the present study. *Holdemanella biformis* was associated with an unhealthy fasting serum lipid profile (Brahe et al., 2015). *Megamonas* had a higher proportion in samples with obesity (Chiu et al., 2014). Some reports showed that the prevalence of *Megamonas* significantly increased in healthy individuals compared with diseased individuals (Jun et al., 2016; Suchodolski et al., 2015). *Dielma fastidiosa* was reported in the human gut (Ramasamy, Lagier, Nguyen, Raoult, & Fournier, 2013). The relative abundance of *Scardovia* was significantly higher in the salivary samples in the group with caries (Zhou et al., 2016). *Scardovia wiggsiae* was associated with the elevated sugar intake (Keller, Kressirer, Belstrøm, Twetman, & Tanner, 2017).

Several studies have shown a difference in gut microbiota between patients with ESRD and normal controls (Vaziri, Wong, et al., 2013; Xu et al., 2017). Improving CKD‐induced dysbiosis can act as a promising targeted treatment in patients with CKD. Recent studies have demonstrated the favorable effect of dietary amylose supplementation on CKD progression and dysbiosis in rats and humans with CKD (Kieffer et al., 2016; Tayebi Khosroshahi et al., 2018; Vaziri et al., 2014). The differential gut microbiota has the potential to guide noninvasive diagnosis and targeted interventions. Given that many factors are associated with the diagnosis of gut microbiota, the biomarker is not a single bacterium but a set of bacteria. The present study found associated phylotypes by analyzing data from the high‐throughput sequencing of the 16S rRNA gene, but the reliability of these bacteria as biomarkers needs to be explored further. Hence, large‐scale prospective studies with extensive sample panels should be conducted to develop more reliable microbiome biomarkers.

## CONFLICT OF INTEREST

The authors declare no conflicts of interest.
